# Use of Synthetic Isoprenoids to Target Protein Prenylation and Rho GTPases in Breast Cancer Invasion

**DOI:** 10.1371/journal.pone.0089892

**Published:** 2014-02-26

**Authors:** Min Chen, Teresa Knifley, Thangaiah Subramanian, H. Peter Spielmann, Kathleen L. O’Connor

**Affiliations:** 1 Markey Cancer Center, University of Kentucky, Lexington, Kentucky, United States of America; 2 Graduate Center for Toxicology, University of Kentucky, Lexington, Kentucky, United States of America; 3 Department of Molecular and Cellular Biochemistry, University of Kentucky, Lexington, Kentucky, United States of America; 4 Department of Chemistry, University of Kentucky, Lexington, Kentucky, United States of America; 5 Center for Structural Biology, University of Kentucky, Lexington, Kentucky, United States of America; Indiana University School of Medicine, United States of America

## Abstract

Dysregulation of Ras and Rho family small GTPases drives the invasion and metastasis of multiple cancers. For their biological functions, these GTPases require proper subcellular localization to cellular membranes, which is regulated by a series of post-translational modifications that result in either farnesylation or geranylgeranylation of the C-terminal CAAX motif. This concept provided the rationale for targeting farnesyltransferase (FTase) and geranylgeranyltransferases (GGTase) for cancer treatment. However, the resulting prenyl transferase inhibitors have not performed well in the clinic due to issues with alternative prenylation and toxicity. As an alternative, we have developed a unique class of potential anti-cancer therapeutics called *P*renyl *F*unction *I*nhibitors (PFIs), which are farnesol or geranyl-geraniol analogs that act as alternate substrates for FTase or GGTase. Here, we test the ability of our lead PFIs, anilinogeraniol (AGOH) and anilinofarnesol (AFOH), to block the invasion of breast cancer cells. We found that AGOH treatment effectively decreased invasion of MDA-MB-231 cells in a two-dimensional (2D) invasion assay at 100 µM while it blocked invasive growth in three-dimensional (3D) culture model at as little as 20 µM. Notably, the effect of AGOH on 3D invasive growth was phenocopied by electroporation of cells with C3 exotransferase. To determine if RhoA and RhoC were direct targets of AGOH, we performed Rho activity assays in MDA-MB-231 and MDA-MB-468 cells and found that AGOH blocked RhoA and RhoC activation in response to LPA and EGF stimulation. Notably, the geranylgeraniol analog AFOH was more potent than AGOH in inhibiting RhoA and RhoC activation and invasive growth. Interestingly, neither AGOH nor AFOH impacted 3D growth of MCF10A cells. Collectively, this study demonstrates that AGOH and AFOH dramatically inhibit breast cancer invasion, at least in part by blocking Rho function, thus, suggesting that targeting prenylation by using PFIs may offer a promising mechanism for treatment of invasive breast cancer.

## Introduction

Breast cancer is the second leading cause of cancer-related deaths among women due to invasion and metastasis [1]. Despite the progress made in prevention, detection, diagnosis and treatment in recent years, more than 70% of breast cancer patients with invasion and metastases still succumb to their disease within 5 years of diagnosis [2]. Therefore, a more effective strategy for treating breast cancer invasion and metastasis is needed.

Dysregulation of small GTPases such as Ras and Rho family GTPases (RhoA, RhoC, Rac1 and Cdc42) is critical to drive the invasion and metastasis of a variety of cancers, including breast carcinomas. Rho small GTPases belong to the Ras superfamily and consist of at least 20 members of 20–30 KDa GTP-binding proteins in mammalian cells [3]. The Rho subgroup of Rho GTPases consists of RhoA, RhoB, and RhoC proteins, which share about 85% amino acid sequence identity [4]. Substantial evidence supports the involvement of aberrant expression of Rho and elevated Rho activity in the metastasis capacity of different types of cancers such as breast, colon, prostate, lung, head and neck, and pancreatic cancers [5]–[8]. Indeed, RhoA and RhoC have been shown to be involved in different stages of tumor progression such as loss of cell polarity and cell junctions, intravasation and vascularization [5].

Like Ras, Rho GTPases act as molecular switches in many cellular processes and cycle from GDP-bound inactive state to the GTP-bound active state. The cycling between these two states is controlled by guanine nucleotide-exchange factors (GEFs), GTPase activating proteins (GAPs), and guanine nucleotide-dissociation inhibitors (GDIs) [3]. To enable this cycle to occur, Rho proteins require a series of post-translational modifications, with the first and most critical step being covalent attachment of an isoprenoid group to the cysteine residue in the carboxyl-terminal CAAX motif (where C represents cysteine, A represents primarily aliphatic amino acids and X represents any amino acid which direct the type of prenylation). This prenylation process includes farnesylation and geranylgeranylation [9]. Specifically, RhoA and RhoC are exclusively geranylgeranylated and RhoB is either geranylgeranylated or farnesylated [9]. Ultimately, active Rho proteins, through binding to their effectors, are involved in a variety of cellular events, including gene regulation, cell cycle progression, migration and transformation [10]. Based on its regulation and functional mode, several strategies targeting Rho signaling modules, such as inhibition of Rho protein-GEF interaction, inactivation of Rho effectors as well as inhibition of lipid modification, have been employed [7]. For example, farnesyltransferase inhibitors (FTI) and geranylgeranyltransferase (GGTase) inhibitors (GGTI) have been used to target Rho prenylation [11], [12]. However, the efficacy, specificity and toxicity of this approach remain a challenge.

We have developed a unique class of potential anti-cancer agents called *P*renyl *F*unction *I*nhibitors (PFIs), which are farnesol or geranylgeraniol analogs that act as alternative substrates for FTase and GGTase. Our previous studies have shown that these unnatural FPP analogs are effective alternative substrates for mammalian FTase. Our lead PFI, anilinogeraniol (AGOH), is the alcohol precursor of 8-anilinogeranyl diphosphate (AGPP), which is incorporated into cellular protein in an FTase and GGTase dependent manner [13], [14]. Notably, in combination with two-dimensional (2D) electrophoresis and immunoblotting, we found that AGOH also labels Rho small GTPases, including RhoA, RhoB and RhoC, in statin treated myeloid leukemia cell lines [14]. These findings prompted us to investigate the use of PFIs to target Rho GTPases as potential therapeutics for the treatment of breast cancer. Here, we report the effect of two lead PFIs, AGOH and AFOH, on breast cancer cell invasion and provide rationale for the use of PFIs in targeting Rho to combat breast cancer.

## Materials and Methods

### Cell Lines and Reagents

**Figure 1:**
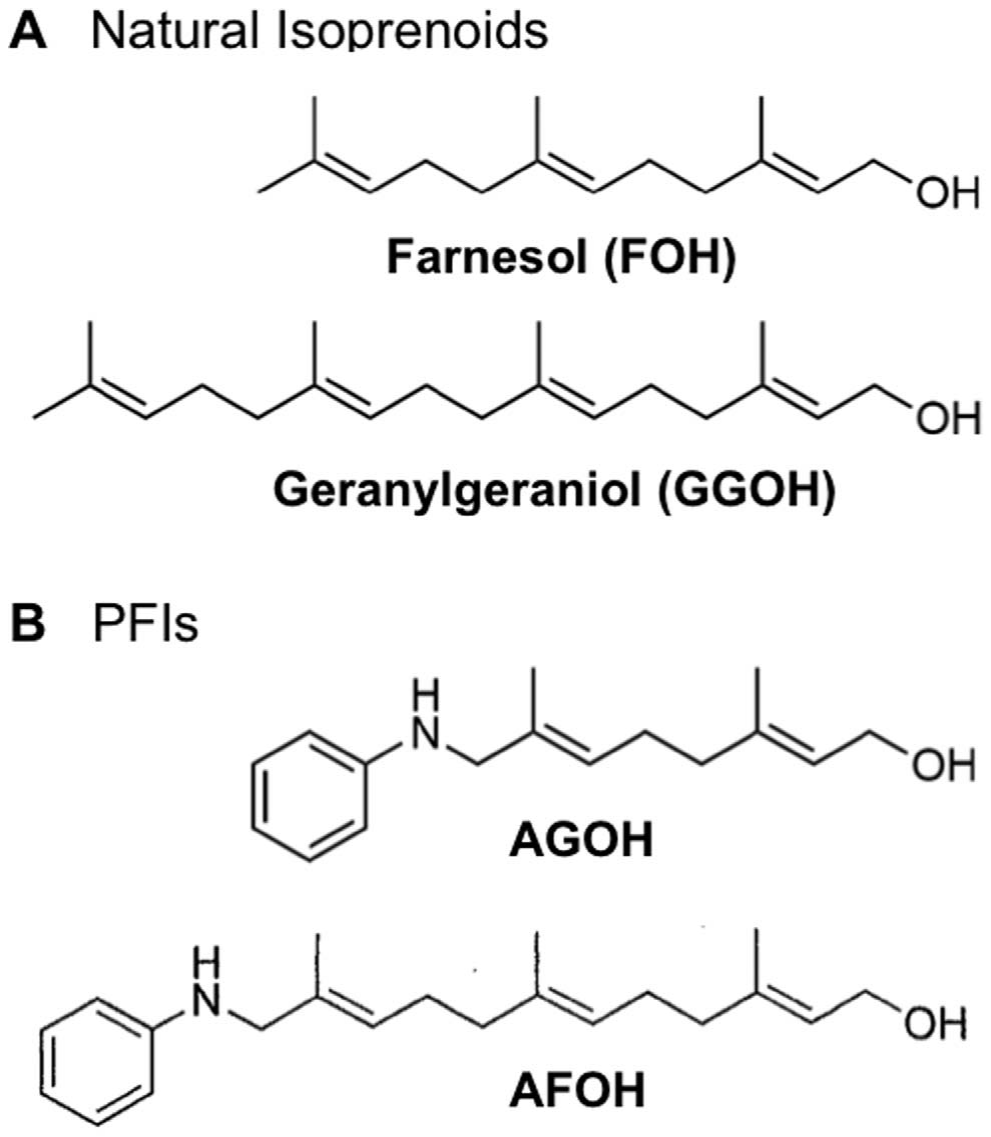
Prenyl Function Inhibitors. Structure relationship between natural isoprenoids (A) and lead PFIs (B).

MDA-MB-231 cells were obtained from American Type Culture Collection (ATCC) and were cultured in low-glucose DMEM. MDA-MB-468 cells [15] were obtained from Janet Price (MD Anderson, University of Texas, Houston, TX) and maintained in DMEM/F12 (1∶1). These two cell lines were cultured with 10% FBS and 1% penicillin/streptomycin/glutamine (Life Technologies). Immortalized non-malignant human breast epithelial MCF10A cell line was obtained from ATCC and maintained in DMEM/F12 media with 5% horse serum supplemented with 20 ng/ml EGF, 10 µg/ml insulin, 0.5 µg/ml hydrocortisone, 100 ng/ml Cholera toxin and 1% penicillin/streptomycin/glutamine. AGOH and AFOH were synthesized as described previously [16], [17]. The relationship between these compounds and the natural isoprenoids is illustrated in Figure 1.

### Matrigel Invasion Assay

Matrigel (10 µg, BD Biosciences) was dried onto the upper well of Transwell chambers (6.5-mm diameter, 8-µm pore size, Corning Life Sciences). One hour before assay, the Matrigel was reconstituted with 100 µl medium and the bottom chamber was coated with 15 µg/ml collagen I. Cells (70% confluent) were trypsinized, rinsed three times with medium plus 250 µg/ml BSA in the presence of AGOH or AFOH at indicated concentration. Cells (5×10^4^) were added to the top wells, and medium/BSA containing 5 ng/ml EGF plus 250 ng/ml insulin was added to the bottom wells; cells were allowed to invade for 5 hrs at 37°C. Non-invaded cells were removed from the top chamber using a cotton swab; invaded cells on the bottom of the Transwell insert were fixed with 100% methanol and stained with 1% crystal violet. Four fields per well were counted and averaged, and the data were presented as the mean number of cells invaded per mm^2^+/− standard deviation from triplicate determinations.

### Three-dimensional (3D) Culture and Immunofluorescence Staining

3D culture for MDA-MB-231 cells and MCF10A cells were performed as described previously [18], [19]. Briefly, MDA-MB-231 cells (5×10^3^) in 200 µl of T4 cell medium (DMEM/F12 medium with 5 ng/ml EGF, 250 ng/ml insulin, 10 µg/ml transferrin, 2.6 ng/ml sodium selenite, 1.4 µM hydrocortisone and 5 µg/ml prolactin) were seeded onto solidified growth factor reduced Matrigel (BD Biosciences; 100 µl per well of 8-well chamber slide) and then covered with 200 µl of 10% Matrigel containing medium. For MCF10A cells, cells (5×10^3^) were resuspended in assay medium (DMEM/F12 medium with 2% horse serum, 5 ng/ml EGF, 10 µg/ml insulin, 1.4 mM hydrocortisone, 100 ng/ml Cholera toxin and 1% penicillin/streptomycin/glutamine) plus 2% Matrigel and plated on the top of solidified growth factor reduced Matrigel. The next day, DMSO, AGOH or AFOH at indicated concentration were added to the cultures and fresh compound added every 4 days for MCF10A cells and every other day for MDA-MB-231 cells. When MCF10A cells were cultured in 3D for 14 days or MDA-MB-231 control cells developed an invasive growth phenotype (approximately 6–8 days), phase contrast images of randomly chosen fields were taken with a Nikon Ti-E inverted microscope using Nikon Elements software. Then 20–40 µl of Matrigel containing colonies for each condition were smeared onto a slide, fixed with 4% paraformaldehyde (for F-actin staining using TRITC-Phalloidin {Sigma}) or cold Methanol/Acetone (1∶1), permeabilized and immunostained using the following antibodies: α6 integrin antibody (rat anti-human CD49f, EMD Millipore), mouse anti-E-cadherin (BD Biosciences), Cy2-conjugated goat anti-rat IgG or Cy3-conjugated donkey anti-mouse IgG (Jackson ImmunoResearch). Slides were mounted in VECTASHIELD mounting medium for fluorescence (Vector Laboratories, Inc.). Confocal images were captured with an Olympus FV1000 confocal microscope using a 60×UPlanS Apo NA 1.35 oil objective and FV10-ASW2 software.

### GST and C3 Protein Electroporation

For C3 exotransferase treatment, MDA-MB-231 cells (3×10^6^) were electroporated at 450 V and 25 µF with 5 µg of bacterially expressed glutathione-S-transferase (GST) or GST-C3 exotransferase purified protein (expression vectors provided by Dr. Keith Burridge, University of North Carolina, Chapel Hill, NC) as described previously [20]. Cells were then incubated with 5 µg/ml polymyxin B nonapeptide hydrochloride (Sigma) for 15 min, rinsed, and then seeded into 3D culture. To test the efficiency and specificity of C3 treatment, cells were harvested for RhoA or Rac1 activity as described below.

### Activity Assays for Small GTPases

GST-Rhotekin Rho binding domain (RBD) binding assay was used to assess Rho activity, as described previously [21], [22]. For these experiments, MDA-MB-231 and MDA-MB-468 cells were grown to 70% confluence while being treated with AGOH, AFOH or DMSO for 3 days. Then, cells were trypsinized, and rinsed with medium plus 250 µg/ml BSA in the presence of AGOH, AFOH or DMSO, as appropriate, at indicated concentrations. Cells (3×10^6^) were plated onto 60-mm dishes coated with collagen I (50 µg/ml in PBS, coated overnight) with the presence of AGOH, AFOH or DMSO for 2 hrs before they were treated with indicated chemoattractants (100 nM LPA {Sigma} or 5 ng/ml EGF {Preprotech}) for 5 min and harvested with Rho lysis buffer (50 mM Tris, pH 7.2, 500 mM NaCl, 1% Triton X-100, 0.25% sodium deoxycholate, 0.1% sodium dodecylsulfate (SDS), 10 mM MgCl_2_, 10 µg/ml protease inhibitor cocktail and 1 mM phenylmethylsulfonyl fluoride). Cleared extracts were incubated for 30 min at 4°C with glutathione beads (GE Healthcare Life Sciences) coupled with GST-Rhotekin RBD fusion protein at 4°C and then washed 3 times with Rho activity assay wash buffer (50 mM Tris, pH 7.2, 150 mM NaCl, 1% Triton X-100, 10 mM MgCl_2_, plus protease inhibitors). Rho content of beads eluents and lysate controls were separated by 15% SDS–polyacrylamide gel electrophoresis (PAGE), transferred to an Immobilon-P^SQ^ PVDF transfer membrane (EMD Millipore) and immunoblotted with mouse anti-RhoA antibody (Santa Cruz Biotechnologies) or mouse anti-RhoC antibody (Abcam).

For Rac and Ras assays, cells were treated as described above. For Rac assays, cells were electroporated with GST or GST-C3 as described above. Cell lysates were harvested with Rac lysis buffer (50 mM Tris, pH 7.4, 100 mM NaCl, 1% NP-40, 10% glycerol, 2 mM MgCl_2_, plus protease inhibitors), clarified by centrifugation, and then incubated with Pak1 Rac/cdc42-binding domain-GST fusion protein bound to glutathione beads for 30 min at 4°C, as described previously [20]. For Ras activity assays, cells were treated with DMSO or 100 µM AGOH as described for the Rho activity assays and then stimulated with EGF. Cell lysates were harvested with Ras lysis buffer (50 mM Tris pH 7.4, 150 mM NaCl, 2.5 mM MgCl, 1% NP-40, 0.5 mM PMSF and 10 µg/ml protease inhibitor cocktail) and incubated with glutathione beads coupled with GST-RalGDS-Ras binding domain-fusion protein at 4°C for 1 hr, as described [23]. For both assays, beads then were washed with lysis buffer for 3 times, and GTP-bound Rac or Ras content of the beads, as well as protein content of lysate controls, were determined by separating proteins by 15% SDS-PAGE and immunoblotting with anti Rac-1 monoclonal antibody (BD Biosciences) or anti-K-Ras antibody (Abcam), respectively.

### Immunoblotting

Cells were treated with PFIs or DMSO solvent control in normal culturing conditions at indicated concentrations for 3 days prior to harvesting with RIPA buffer containing protease inhibitors. Total cell lysates (80 µg) were separated by 15% SDS-PAGE and probed with rabbit sera against the anilinogeranyl moiety as describe previously [13]. For p-MLC assay, cells were plated in collagen I coated dishes for 2 hrs before stimulation with 100 nM LPA for 5 min. Then cells were harvested in RIPA buffer with phosphatase inhibitors (150 mM NaCl, 0.5 mM EGTA, 0.5% sodium deoxycholate, 0.1% SDS, 1% Triton X-100, 50 mM Tris-HCl pH 7.4, 15 µg/ml protease inhibitor cocktail, 1 mM PMSF, 50 mM NaF and 10 mM sodium pyrophosphate). Then, total cell lysates (80 µg) were subjected to 15% SDS-PAGE, transferred to PVDF membrane and immunoblotted with p-MLC S19 (Cell Signaling Technology) or total myosin light chain 2 antibody (Cell Signaling Technology). β-actin (monoclonal antibody; Sigma) was used as the loading control.

## Results

### Lead PFI Compound AGOH Inhibits MDA-MB-231 Cell Invasion

**Figure 2:**
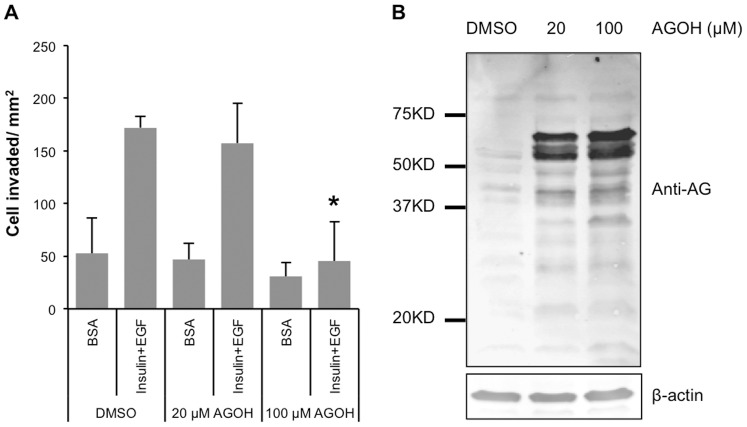
AGOH inhibits MDA-MB-231 cells invasion. (A) MDA-MB-231 cells were treated with AGOH at the indicated concentration and then assessed for Matrigel invasion toward the combination of 5 ng/ml EGF and 250 ng/ml insulin. (B) Cells treated with AGOH for 3 days were harvested in RIPA buffer, immunoblotted with polyAG-antibody. Asterisk (*) symbolizes a p value <0.05. These results are representative from at least three separate experiments.

Previous studies demonstrated that AGOH is a pro-drug version of the FPP analog AGPP and is incorporated into normally prenylated cellular proteins in an FTase and GGTase dependent manner [14], [16], [24], [25]. Growth factors such as EGF and insulin are critical for mammary gland morphogenesis, serve as the important survival factors of mammary epithelial cells [26], and are used in 3D invasive growth assays [18], [27]. We sought to make the invasion assay as close as possible to the 3D assay and tested the effects of components of the T4 medium (used for 3D culture) in the transwell invasion assay. We observed that the combination of EGF and insulin displayed a synergistic effect on the invasive potential of MDA-MB-231 cells and closely mimicked results from the complete T4 medium (data not shown). To determine how PFIs impact invasion, we treated MDA-MB-231 cells in 2D culture with 20 µM or 100 µM AGOH for 3 days, then performed Matrigel invasion assays toward the combination of 5 ng/ml EGF plus 250 ng/ml insulin. We found that AGOH inhibited invasion of MDA-MB-231 cells at concentration of 100 µM compared to control cells. In contrast, at 20 µM, AGOH did not have significant effects (Figure 2A). To confirm AGOH was incorporated into proteins, MDA-MB-231 cell lysates were immunoblotted for anilinogeranyl (AG) modified cellular proteins using a high affinity rabbit anti-sera specific for the anilinogeranyl moiety (Figure 2B) [13]. Consistent with previous studies [28], [29], we found that a broad range of bands (molecular weight from 17–100 KD) correlated well with known prenylated proteins appeared in the AGOH treated samples compared to DMSO control. In addition, the pattern of mid-range molecular weight proteins that incorporated AGOH correspond to farnesylated nuclear lamins (Figure 2B), similar to those found in other cell types [28].

### AGOH Inhibits MDA-MB-231 Cells Invasive Growth in 3D Culture

**Figure 3:**
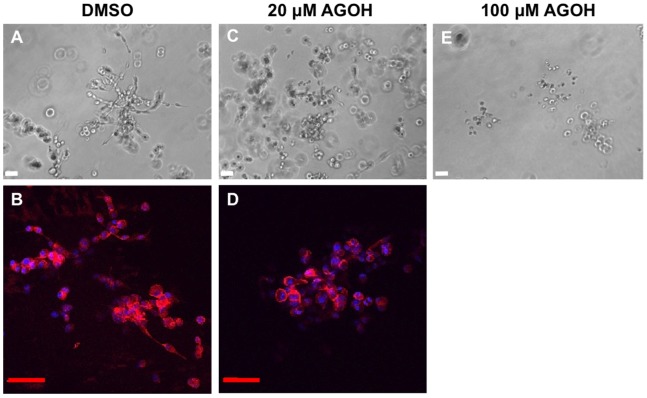
AGOH inhibits 3D invasive growth of MDA-MB-231 cells. MDA-MB-231 cells were seeded in Matrigel and treated with AGOH at the indicated concentration. After culturing for 8 days, phase contrast images were taken from randomly chosen fields (A, C, E) or Matrigel containing colonies were fixed and immunostained for F-actin (phalloidin, red) and nuclei (DAPI, blue) (B, D). The representative images from three separate experiments are shown. Scale bars represent 50 µm.

Culturing cells in 3D is commonly used to assess the physiologically relevant morphogenesis and invasive potential of breast epithelial and cancer cells [18], [30]. Accordingly, we next investigated the effect of AGOH on 3D invasive growth. In contrast to the 2D invasion assay, treatment of MDA-MB-231 cells in 3D culture with 20 µM AGOH showed a significant reduction in invasive growth (Figure 3A and C). This effect is even more dramatic at 100 µM where live colonies were difficult to find (Figure 3E). The immunocytochemistry staining revealed that DMSO-treated cells developed invasive growth (Figure 3B); however, cells treated with 20 µM AGOH lost their invasive potential in 3D culture (Figure 3D). Surprisingly, Ras activities in these cells were not affected (Figure S1), suggesting that Ras was not the primary target for AGOH in these breast cancer cells.

### Rho GTPases are Required for MDA-MB-231 Cells Invasive Growth

**Figure 4:**
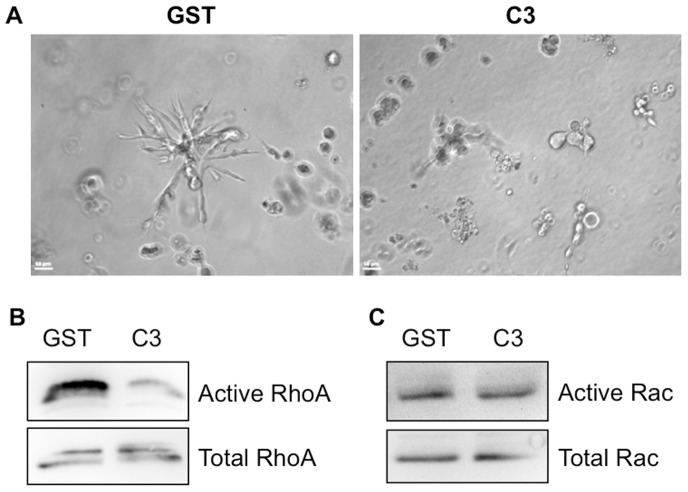
Rho GTPases are required for the invasive growth of MDA-MB-231 cells. Cells were electroporated with 5 µg GST or GST-C3 exotransferase, then seeded in 3D Matrigel and imaged (A) as described in Figure 3, or assessed for RhoA (B) and Rac1 (C) activities as described in the Methods section.

Tumor invasive growth is a complex, multistep program involving in the interplay of tumor cells and the microenvironment, and in turn tumor cells acquiring the propensity for migration, invasion and proliferation [31]. Rho signaling is engaged in at least two distinct types of motility in 3D matrix: Rho/ROCK-dependent amoeboid motility and Rac-dependent mesenchymal motility [32]. To test whether Rho is required for the invasive growth of MDA-MB-231 cells, cells were electroporated with 5 µg GST or GST-C3 exotransferase, which inactivates RhoA, RhoB and RhoC by ADP ribosylation. These results demonstrated that C3 treatment phenocopies AGOH treatment (Figure 4A), suggesting that Rho GTPases are required for the invasive growth of MDA-MB-231 cells. Cells were also assayed for Rho (Figure 4B) and Rac (Figure 4C) activities to ensure the efficiency and specificity of C3 on Rho activity. The activity assays showed that C3 efficiently blocked active Rho but had no effect on Rac activity. These data suggest that AGOH may affect the invasive potential of MDA-MB-231 cells by modifying Rho signaling.

### Rho Activity is Inhibited by AGOH

**Figure 5:**
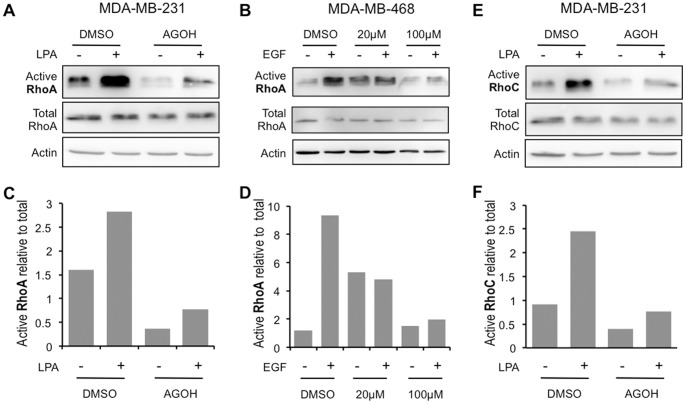
AGOH blocks RhoA and RhoC activation in response to LPA. MDA-MB-231 cells (A and E) or MDA-MB-468 cells (B) were treated with 100 µM AGOH for 3 days, plated on collagen I coated dishes in the presence of PFI and then treated with 100 nM LPA or 5 ng/ml EGF as indicated for 5 min before harvesting for RhoA (A and B, quantified in C and D) or RhoC (E, quantified in F) activity assay. Rho activity assays are representative from at least three separate experiments.

To test whether Rho activity is affected by AGOH, we performed GST-Rhotekin RBD pull down assay to assess the activity of Rho. We treated MDA-MB-231 cells with AGOH for 3 days, then cells were seeded onto collagen-coated dishes for 2 hrs in the presence of 100 µM AGOH before treatment with 100 nM LPA for 5 min. Results from the Rho activity assay showed that LPA stimulated Rho activity; however, AGOH at 100 µM significantly blocked active RhoA to the basal level (Figure 5A and C). We also found that AGOH inhibited RhoA activity in MDA-MB-468 cells in response to 5 ng/ml EGF (Figure 5B and D), thus confirming that this effect was not cell type or growth factor specific. In addition, RhoA activity of MDA-MB-231 cells treated with AGOH and stimulated with 10% serum provided similar results (data not shown), further supporting the inhibitory effect of AGOH on Rho.

The Rho subgroup contains three isoforms: RhoA, RhoB and RhoC. Previous studies demonstrated that RhoB is poorly expressed in MDA-MB-231 cells [33]. RhoC plays an important role in tumor metastasis [8], [34] and is considered a biomarker for invasive breast cancer [35]. To determine the effect of AGOH on RhoC, we immunoblotted GST-Rhotekin RBD pull down assay with an anti-RhoC antibody. Our results demonstrated that AGOH also blocked the activity of RhoC in response to LPA in MDA-MB-231 cells (Figure 5E and F). Taken together, the data support a mechanism by which AGOH inhibits the invasive potential of breast cancer cells, at least in part, through inhibition of RhoA and RhoC activity.

### AFOH is more Potent than AGOH in Inhibiting Rho Activity and 3D Invasive Growth

AGOH enters cells through the plasma membrane and is phosphorylated in sequential kinase reactions leading to AGPP, which is a substrate analog of FPP preferentially utilized by FTase [13]. Notably, RhoA and RhoC are normally geranylgeranylated by GGTase-1. We have found that AGPP can also be converted to AFPP, a substrate analog of GGPP preferentially utilized by GGTase I to modify cellular proteins [36]. We hypothesized that treatment with the geranylgeraniol analog AFOH would be more potent at inhibiting Rho and breast carcinoma invasion than AGOH.

**Figure 6:**
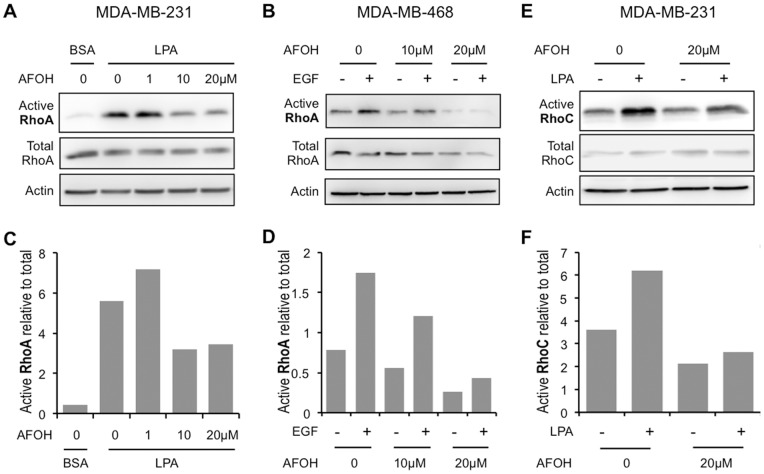
AFOH blocks RhoA and RhoC activation in response to LPA or EGF. MDA-MB-231 cells (A, C, E and F) or MDA-MB-468 cells (B and D) were treated with AFOH at the indicated concentration for 3 days, plated on collagen I coated dishes in the presence of AFOH and then treated with 100 nM LPA (A and E, quantified in C and F) or 5 ng/ml EGF (B, quantified in D) for 5 min prior to harvesting for RhoA (A–D) or RhoC (E, F) activity assays. Rho activity assays are representative from at least three separate experiments.

**Figure 7:**
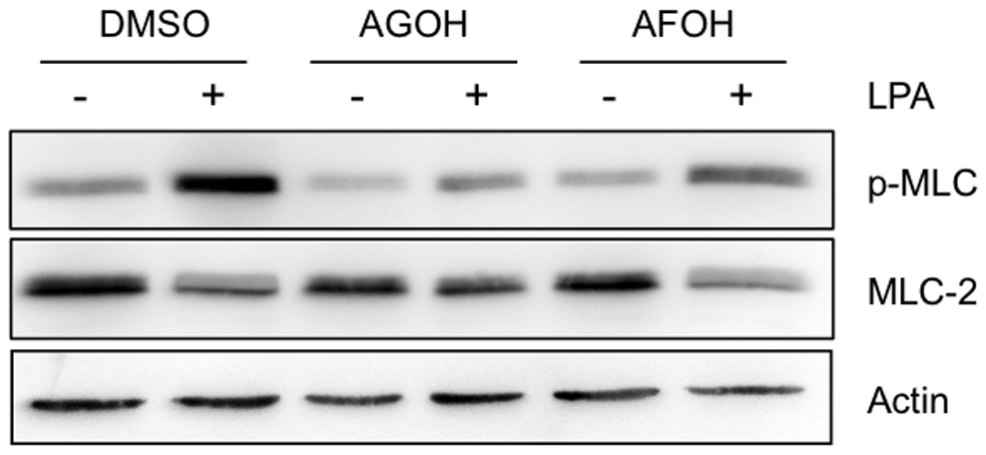
PFIs block p-MLC downstream of Rho signaling in MDA-MB-231 cells. MDA-MB-231 cells were treated with 100 µM AGOH and 20 µM AFOH for 3 days, plated on collagen I coated dishes, and then treated with 100 nM LPA for 5 min. Cell lysates were immunoblotted with p-MLC. Total MLC-2 and β-actin serve as the loading controls.

**Figure 8:**
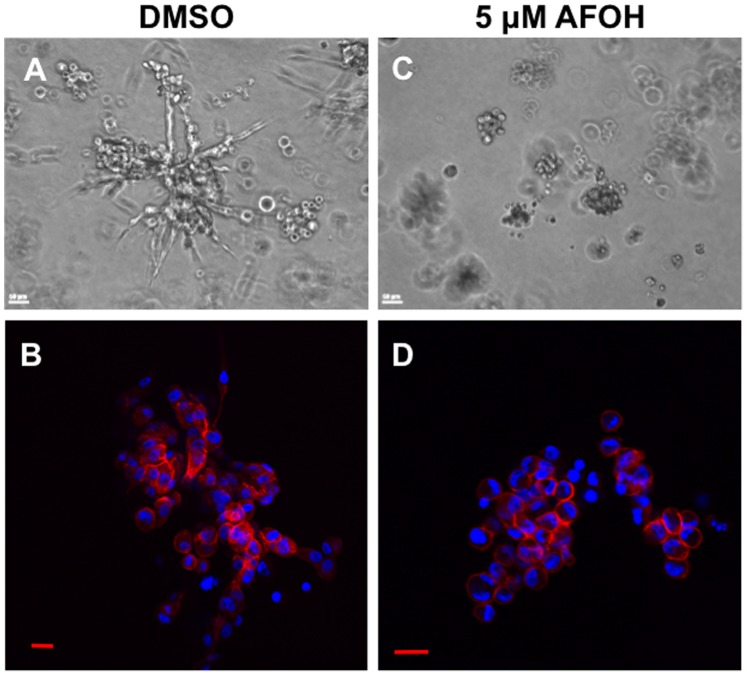
AFOH blocks 3D invasive growth of MDA-MB-231 cells. Cells were seeded in growth factor reduced Matrigel, treated with DMSO (A, B) or 5 µM AFOH (C, D) for 10 days. Then phase contrast images were taken from randomly chosen fields (A, C). Colonies grown in Matrigel were smeared onto slides, immunostained for F-actin (red) and nuclei (blue), and then imaged by confocal microscopy (B, D). The representative images from three experiments are shown.

To test this concept, MDA-MB-231 cells were treated with various concentrations of the geranylgeraniol analog AFOH for 3 days prior to assessing Rho activity. As shown in Figure 6A and C, RhoA activity stimulated by LPA displayed a dose-dependent inhibition with AFOH treatment. AFOH inhibited RhoA at concentrations as low as 10 µM, which was 10 times more potent compared to AGOH under the same conditions (Figure 5A and C). The similar potency was also observed in MDA-MB-468 cells in response to EGF (Figure 6B and D). Importantly, RhoC activity was also inhibited (Figure 6E and F). To confirm RhoA and RhoC activities were inhibited by PFIs, we further tested the level of phosphorylation of myosin light chain (p-MLC) downstream of Rho/ROCK signaling pathway. As shown in Figure 7, AGOH and AFOH significantly blocked the phosphorylation of MLC in MDA-MB-231 cells in response to LPA stimulation. Accordingly, MDA-MB-231 cells treated with AFOH in 3D culture showed a dramatic inhibition of invasive growth at concentrations between 1 µM to 5 µM (Figure 8A–D). Collectively, the data further demonstrated that RhoA and RhoC are targets of PFIs in breast cancer cells.

### The Lead PFIs AGOH and AFOH do not Affect MCF10A 3D Growth

**Figure 9:**
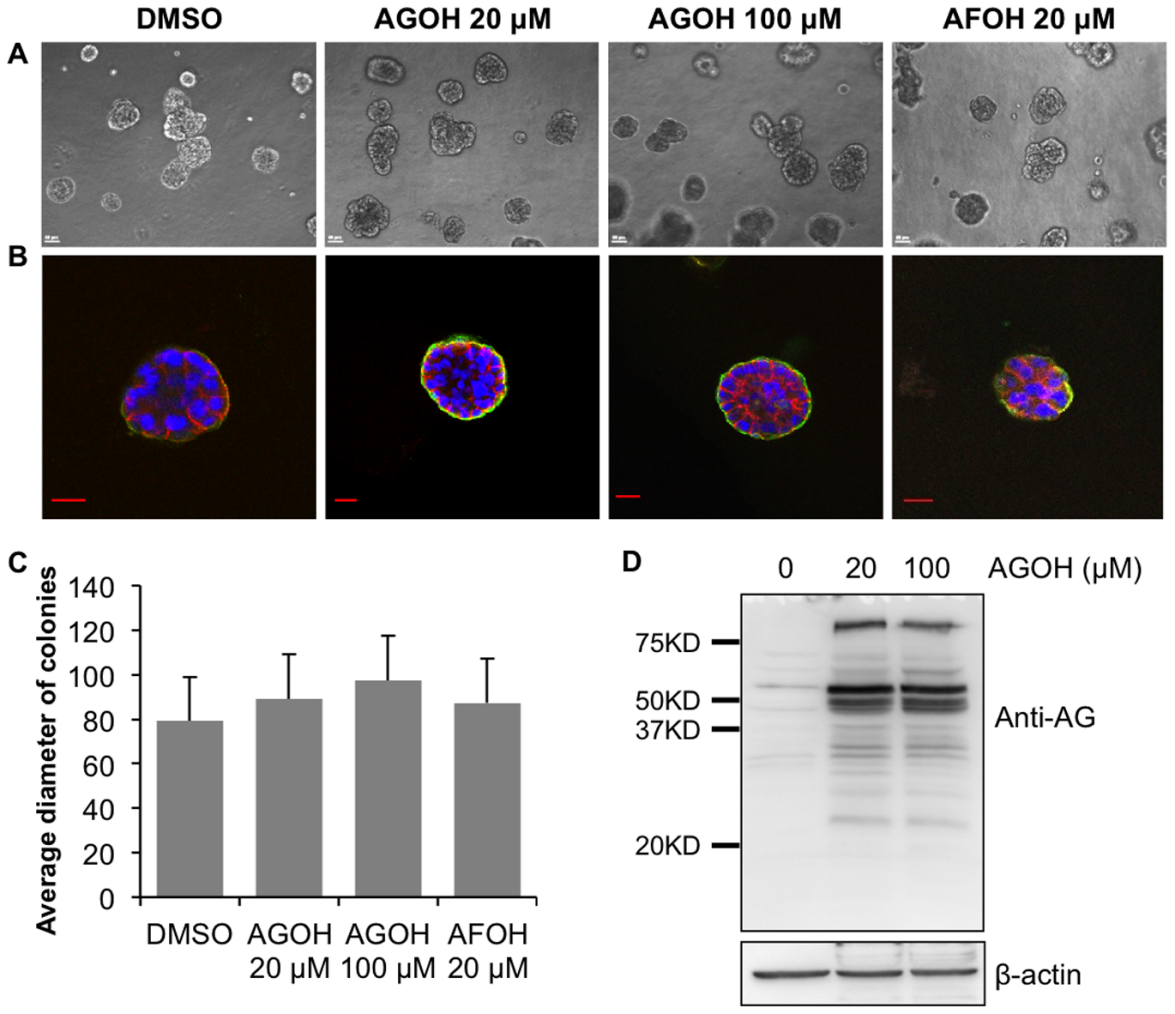
AGOH and AFOH do not affect 3D growth of MCF10A cells. MCF10A were seeded in Matrigel and treated with AGOH and AFOH at the indicated concentrations for 14 days. Then the phase contrast images (A) were taken from randomly chosen fields. Matrigel-containing colonies were smeared and immunostained for integrin α6 (green), E-cadherin (red) and nuclei (blue) (B). Diameter of 60–80 individual colonies was measured and averaged in each condition (C). Cells were treated with AGOH for 3 days, harvested in RIPA buffer, and immunoblotted with polyAG antibody (D). Representative images from at least three separate experiments are shown. Scale bars in phase contrast equal 50 µm and in confocal images represent 20 µm.

To test whether these drugs have any toxicity to non-malignant cells, we treated MCF10A cells in 3D culture with the indicated PFIs starting on Day 1 until harvested on Day 14. As shown in Figure 9, DMSO-treated control MCF10A cells formed round, small, well-organized acinar-like structures. Interestingly, AGOH and AFOH treatment did not produce significant difference in terms of colony size, organization of acinar structures or the overall number of colonies (Figure 9A–C). Notably, AGOH was incorporated efficiently into MCF10A cellular proteins (Figure 9D) at levels similar to MDA-MB-231 cells, suggesting that this effect was not due to the lack of efficiency of PFIs incorporation in MCF10A cells. We concluded from these experiments that our lead PFIs, especially AFOH, were able to target Rho and block invasive growth of malignant breast cancer cells with minimal toxicity to the normal cells.

## Discussion

Rho signaling is important for actin cytoskeleton reorganization, focal adhesion formation, and cell migration. Accordingly, Rho proteins, in particular RhoA and RhoC, play critical roles in tumor invasion and metastasis in a variety of cancers including breast cancer [37]–[40]. Therefore, RhoA and RhoC are considered to be valuable therapeutic targets. Rho proteins, however, have not been considered “druggable” by conventional therapeutics since the Ras superfamily of small GTPases are globular in structure with limited surface sites suitable for small molecule binding [41]. Therefore, current strategies for targeting Rho involve interfering upstream regulators as well as downstream effectors of Rho signal transduction. These strategies include inhibition of Rho prenylation by blocking the mevalonate pathway with statins [42], [43] or with by FTIs and/or GGTIs [12], directly suppressing Rho by siRNA or bacterial toxin C3, and blocking downstream effectors such as ROCK [7], [44]. A recently developed strategy involves the small molecule Rhosin, which interferes with the interaction of Rho and its GEF [45]. Of these strategies, only inhibition of protein prenylation has been tested clinically for the treatment of cancer.

Prenylation of Rho and most other Ras superfamily small GTPases is obligatory for their biological function. Targeting protein prenylation has received considerable attention, which resulted in the generation of FTIs and GGTIs. Initial FTI clinical trials in breast cancer showed substantial responsiveness; however, notable toxicity and the inability of FTIs to yield clear survival benefits halted these trials. The lack of FTI efficacy is attributed to alternative prenylation of proteins (such as K-Ras) by GGTase-I when FTase is inhibited and their inability to block geranylgeranylated proteins, such as Rho GTPases [43].

To circumvent these hurdles, we developed the PFI class of unnatural synthetic isoprenoids that act as the alternative FTase and/or GGTase substrates. The lead PFIs, AGOH and AFOH, are pro-drug structural analogs of the respective preferred substrates for FTase (FPP) and GGTase (GGPP). In cells, the PFIs are converted to their corresponding diphosphates and are transferred by FTase and GGTase to CAAX substrates. In MDA-MB-231 cells, PFIs inhibited invasion in both 2D and 3D and blocked RhoA and RhoC activation in response to growth factors. Moreover, the PFIs showed no overt cytotoxicity in non-malignant MCF10A cells at concentrations that blocked colony formation and invasive growth of the MDA-MB-231 cells in 3D culture. Thus, the PFIs, such as AGOH and AFOH, represent a new class of molecules for the inhibition of Rho proteins that can be further developed as potential anti-metastatic agents.

In our study, we uncovered multiple variables that impact how PFIs could be screened, which have important implications for drug development. First, we found that cells grown under the more physiological 3D condition are more sensitive to PFIs than in 2D culture condition. This observation indicates that using 2D cell culture for library screening could miss potentially effective compounds based on the lack of proper physiological context and cellular architecture, as has been previously suggested [46]–[48]. Second, the lead geranylgeraniol derivative AFOH is more potent than the farnesol derivative AGOH in inhibiting the invasive potential of MDA-MB-231 cells and blocking the activation of RhoA and RhoC in response to growth factors. These differences are likely due to the need for AGOH to be elongated to AFOH, which occurs with low efficiency, in order for it to be an efficient GGTase substrate [36]. In contrast, AFOH acts as a more direct substrate for GGTase. The enhanced potency of AFOH may be due to the greater importance of geranylgeranylated proteins compared to farnesylated proteins in breast cancer cell invasion. Third, these two lead compounds did not exhibit significant toxicity for MCF10A cells in 3D culture, even at concentrations that completely inhibited invasive growth of MDA-MB-231 cells. This finding is particularly interesting considering the challenge of developing drugs that target Rho with acceptable toxicity. Our data showed that this effect is not due to the failure of PFIs to incorporate into cellular proteins in MCF10A cells. A better explanation for the lack of impact of PFIs on MCF10A cells is that RhoA and RhoC are dispensable for acinar formation and cell growth control in non-malignant cells such as MCF10A. In support of the concept that Rho proteins are needed for the malignant transformation of MCF10A cells, a recent study demonstrated that overexpression of constitutively active RhoC G14V in MCF10A cells increased lung metastasis in mice [49].

We demonstrated that RhoA and RhoC are PFI targets that are essential for the invasive potential of breast cancer cells. Our data (Figure 4) clearly verified that Rho proteins are critical for the invasive potential of MDA-MB-231 cells in 3D culture, which is in accordance with other studies [50], [51]. However, we cannot rule out the possibility that PFI activity is also due to the involvement of other geranylgeranylated proteins. The Ras family proteins such as Rap and Ral are also geranylgeranylated. In fact, Ral signaling has been shown to be important in the regulation of LPA–mediated migration and invasion in MDA-MB-231 cells [52]. In addition, we found that RalA and RalB were labeled with anti-AG polyclonal antibody in myeloid leukemia cell lines by using 2D electrophoresis in conjunction with immunoblotting [14]. Therefore, it is likely that RhoA and RhoC represent a subset of proteins important for tumor cell invasion that are affected by PFIs and that protein prenylation in general remains the primary target of PFIs.

In summary, our study demonstrates that hijacking the activity of FTase and GGTase to modify proteins with alternative prenyl donors is a viable strategy for targeting Rho activity in cells that is effective in blocking the invasive potential of breast carcinoma cells. Therefore, our results validate the concept that PFIs provide a promising mechanism of targeting Rho for the treatment of breast cancer. The discovery that PFIs have anti-Rho activity provides new impetus to target and block the function of protein prenyl groups.

## Supporting Information

Figure S1 **AGOH does not affect Ras activity.** MDA-MB-231 cells were treated with 100 µM AGOH or DMSO for 3 days, plated on collagen I coated dishes, and then treated with 5 ng/ml EGF for 5 min in the presence of AGOH or DMSO, as indicated, prior to harvesting for K-Ras activity, as described in the Materials and Methods section.
(TIFF)
