# Supplementary figures and images for: Use of Synthetic Isoprenoids to Target Protein Prenylation and Rho GTPases in Breast Cancer Invasion
Source: PLoS One. 2014 Feb 26;9(2):e89892. doi: 10.1371/journal.pone.0089892 (PMC3935959; doi:10.1371/journal.pone.0089892)

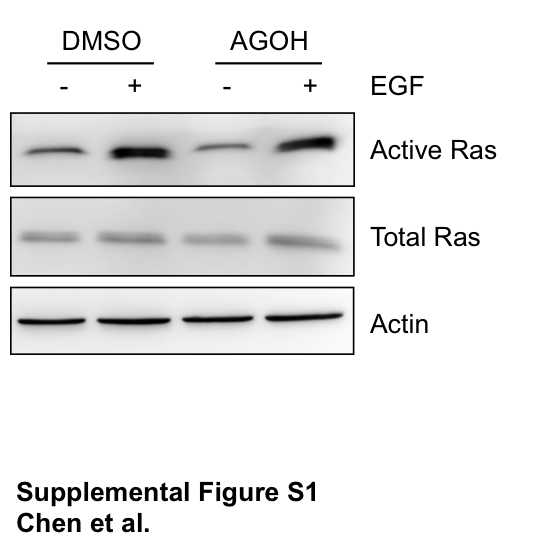

Supplement: Figure S1 — AGOH does not affect Ras activity. MDA-MB-231 cells were treated with 100 µM AGOH or DMSO for 3 days, plated on collagen I coated dishes, and then treated with 5 ng/ml EGF for 5 min in the presence of AGOH or DMSO, as indicated, prior to harvesting for K-Ras activity, as described in the Materials and Methods section. (TIFF) [file pone.0089892.s001.tiff]
